# FACTORS THAT IMPACT SUCCESSFUL AGING AMONG OLDER ADULTS LIVING WITH HIV/AIDS

**DOI:** 10.1093/geroni/igac059.1041

**Published:** 2022-12-20

**Authors:** Erin Robinson, Molly Perkins, Alexis Bender

## Abstract

The past two decades have brought significant medical advances in antiretroviral therapies (ART) for people living with HIV/AIDS (PLWHA). This has enabled PLWHA to live longer and healthier lives than ever before. In fact, nearly half of all PLWHA in the United States are age 50+ and HIV is treated as a chronic disease, instead of a terminal illness. Despite these advancements older adults living with HIV/AIDS (OALWHA) are still a highly stigmatized population who faces challenges with their health and overall well-being. This symposium will highlight recent research on factors that play a role in successful aging among OALWHA. Our first presentation leverages a quantitative dataset of OALWHA and examines the relationship between childhood sexual abuse and ART adherence, with a particular focus on substance abuse as a potential mediator. Our second presentation features a qualitative study with OALWHA that explores their conceptions of successful aging. Our third presentation includes a sample of medical case managers who serve OALWHA and examines the feasibility of using a cognitive screening tool with this population. Cognitive screening with OALWHA can help provide early indicators of cognitive decline and initiate early intervention. Finally, our fourth presentation includes a sample of older women living with HIV/AIDS and examines their support needs and resources, particularly to help understand their complex caregiving needs. Findings from these four studies advance our understanding of OALWHA and their needs to ensure continued successful aging. This symposium informs further research and direct practice with this population.

